# A Two-Faced Gut Microbiome: Butyrogenic and Proinflammatory Bacteria Predominate in the Intestinal Milieu of People Living with HIV from Western Mexico

**DOI:** 10.3390/ijms25094830

**Published:** 2024-04-29

**Authors:** Tonatiuh Abimael Baltazar-Díaz, Jaime F. Andrade-Villanueva, Paulina Sánchez-Álvarez, Fernando Amador-Lara, Tania Holguín-Aguirre, Karina Sánchez-Reyes, Monserrat Álvarez-Zavala, Rocío Ivette López-Roa, Miriam Ruth Bueno-Topete, Luz Alicia González-Hernández

**Affiliations:** 1 Instituto de Investigación en Enfermedades Crónico-Degenerativas, Departamento de Biología Molecular y Genómica, Centro Universitario de Ciencias de la Salud, Universidad de Guadalajara, Sierra Mojada 950, Guadalajara 44340, Mexico; tonatiuhabd@gmail.com; 2 Instituto de Investigación en Inmunodeficiencias y VIH, Departamento de Clínicas Médicas, Centro Universitario de Ciencias de la Salud, Universidad de Guadalajara, Hospital 278, Guadalajara 44280, Mexico; drjandradev@gmail.com (J.F.A.-V.); karina_reyesqfb@hotmail.com (K.S.-R.); monse_belan@hotmail.com (M.Á.-Z.); 3 Unidad de VIH, Hospital Civil de Guadalajara Fray Antonio Alcalde, Hospital 278, Guadalajara 44280, Mexico; pausanchezalvarez@gmail.com (P.S.-Á.); fernando.amador@academicos.udg.mx (F.A.-L.); taniaholguinaguirre@gmail.com (T.H.-A.); 4 Laboratorio de Investigación y Desarrollo Farmacéutico, Centro Universitario de Ciencias Exactas e Ingenierías, Universidad de Guadalajara, Marcelino García Barragán 1421, Guadalajara 44430, Mexico; rocio.lopez@academicos.udg.mx

**Keywords:** HIV infection, gut microbiota, dysbiosis, SCFA, butyrate

## Abstract

HIV infection results in marked alterations in the gut microbiota (GM), such as the loss of microbial diversity and different taxonomic and metabolic profiles. Despite antiretroviral therapy (ART) partially ablating gastrointestinal alterations, the taxonomic profile after successful new ART has shown wide variations. Our objective was to determine the GM composition and functions in people living with HIV (PLWHIV) under ART in comparison to seronegative controls (SC). Fecal samples from 21 subjects (treated with integrase strand-transfer inhibitors, INSTIs) and 18 SC were included. We employed 16S rRNA amplicon sequencing, coupled with PICRUSt2 and fecal short-chain fatty acid (SCFA) quantification by gas chromatography. The INSTI group showed a decreased α-diversity (*p* < 0.001) compared to the SC group, at the expense of increased amounts of *Pseudomonadota* (*Proteobacteria*), *Segatella copri*, *Lactobacillus*, and Gram-negative bacteria. Concurrently, we observed an enrichment in *Megasphaera* and *Butyricicoccus*, both SCFA-producing bacteria, and significant elevations in fecal butyrate in this group (*p* < 0.001). Interestingly, gut dysbiosis in PLWHIV was characterized by a proinflammatory environment orchestrated by *Pseudomonadota* and elevated levels of butyrate associated with bacterial metabolic pathways, as well as the evident presence of butyrogenic bacteria. The role of this unique GM in PLWHIV should be evaluated, as well as the use of butyrate-based supplements and ART regimens that contain succinate, such as tenofovir disoproxil succinate. This mixed profile is described for the first time in PLWHIV from Mexico.

## 1. Introduction

Antiretroviral therapy (ART) in people living with HIV (PLWHIV) has markedly increased life expectancy among these individuals [1]. The success of ART is reflected in the fact that HIV infection has become a chronic disease. Consequently, the clinical approach has significantly turned towards different comorbidities related to the effect of different ART regimens and other classical factors, such as the style of life, cardiovascular disease, metabolic disorders, hepatic and renal disease, and aging [2,3]. During the early phase of HIV infection, the gut-associated lymphoid tissue (GALT) suffers from a significant depletion of CD4^+^ Th17 lymphocytes, resulting in damage and dysfunction of the gastrointestinal system [4]. This damage to the intestinal epithelium occurs earlier than alterations in CD4^+^ lymphocytes at the systemic level [5]. This set of observed alterations, together with a low CD4^+^ nadir, may predict the time required to achieve undetectability and immunocompetence in patients under ART [6,7]. Although ART has been highly effective in achieving virological suppression, both restoring peripheral CD4^+^ T lymphocytes and reducing their activation [8], it was shown that the restoration of these lymphocytes in the lamina propria is incomplete. Individuals that fail to restore a normal CD4^+^ cell count are prone to morbidity and mortality, including malignancies and cardiovascular events [9]. Even under long-term ART [4], it is not possible to eradicate HIV from reservoirs where viral replication persists [10], such as GALT.

The gut microbiota (GM) comprises a complex ecosystem of diverse microorganisms, mostly bacteria, from whose proper balance with the host benefits are obtained (“eubiosis”). Conversely, when the microbiota loses its mutualistic association with the host, dysbiosis occurs, which is characterized by a loss of bacterial diversity and metabolic functions [11,12]. HIV infection is reported to cause dysbiosis in the GM [13]. Contrarywise, it was observed that ART increases bacterial diversity and improves some systemic inflammation markers [14]. Interestingly, this does not occur in all populations, and in some cases, many of the dysbiosis features are maintained [15]. Alterations in the GM profile are mirrored in the metabolites produced by gut bacteria. Among these metabolites, SCFAs (short-chain fatty acids) are particularly important. These SCFAs (acetic, propionic, and butyric acids) are produced especially by members of the *Bacillota* (previously known as *Firmicutes*) phylum [16]. Butyric acid plays an essential role as an energy source for enterocytes, helping to maintain the integrity of the intestinal barrier and regulating the mucosa-associated immune system [17,18]. In this sense, evidence shows that in gastrointestinal inflammatory conditions, such as Crohn’s disease, butyrate supplementation reduces inflammation [19]. Delving into its immunomodulatory properties, it was shown that the concentration of SCFAs correlates with the number of regulatory T cells, involved in suppressing the inflammatory response. Consequently, SCFAs play an important role in defining the anti-inflammatory or pro-inflammatory environment in the intestine [20]. One of the fundamental mechanisms by which butyrate and other SCFAs modulate the inflammatory response is their potent global inhibition activity (“pan-inhibition”) of histone deacetylases (HDACs) [18,21], which could be related to the observed pleiotropic effect of SCFAs [22].

Despite the alterations described by multiple studies, which reported decreases in commensal bacteria such as *Bacillota* and the loss of bacterial diversity [23,24,25], these traits do not seem to be generalizable. Previous studies from China and Colombia showed that a butyrogenic microbiota predominates in PLWHIV [26,27]. In view of this evidence, environmental factors that influence the composition of the microbiota, particularly those that can shape pro-inflammatory profiles, become relevant. These factors, such as a high-fat and high-carbohydrate diet plus genetic predispositions towards metabolic disorders, converge in the Mexican population [28,29]. This is of particular concern in the current HIV context, especially given the evidence indicating an increase in cardiometabolic events under integrase strand-transfer inhibitor-based (INSTI-based) ART [30].

In the present study, we analyzed the GM of PLWHIV from western Mexico under INSTI-based ART and virological suppression, in comparison with that of a clinically healthy seronegative population. In addition to determining the differential taxa of each group and quantifying effector metabolites, such as SCFAs, we describe new bacterial metabolic pathways associated with a particular butyrogenic profile of PLWHIV. To our knowledge, these results constitute new findings in the Mexican population, contributing to understanding the implications of the GM in chronic HIV infection.

## 2. Results

### 2.1. Cross-Sectional Study, Clinical and Biochemical Characteristics

A total of thirty-nine participants were recruited. Twenty-one patients were in the INSTI group, eighteen patients (85.7%) were under bictegravir (BIC) treatment, and three patients (14.3%) were receiving dolutegravir (DTG). Eighteen seronegative healthy controls were in the SC group. The clinical characteristics of the participants are showed in Table 1. No significant differences were found between the groups in terms of age, gender, and BMI. The INSTI group presented risk factors such as smoking (33.3%, *n* = 7) and alcohol use (61.9%, *n* = 13), as well as comorbidities associated with the use of INSTIs (osteoporosis, 28.6%, *n* = 6, and one patient with borderline personality disorder, Supplementary Table S1). The INSTI group showed a slightly elevated concentration of serum AST compared with the SC group (31.82 vs. 22.89 IU/L, respectively, *p* = 0.039) and a decreased concentration of HDL-c (39.19 mg/dL vs. 48.1 mg/dL, *p* = 0.025, Supplementary Table S1).

### 2.2. Gut Microbiota Diversity and Structure

**Figure 1:**
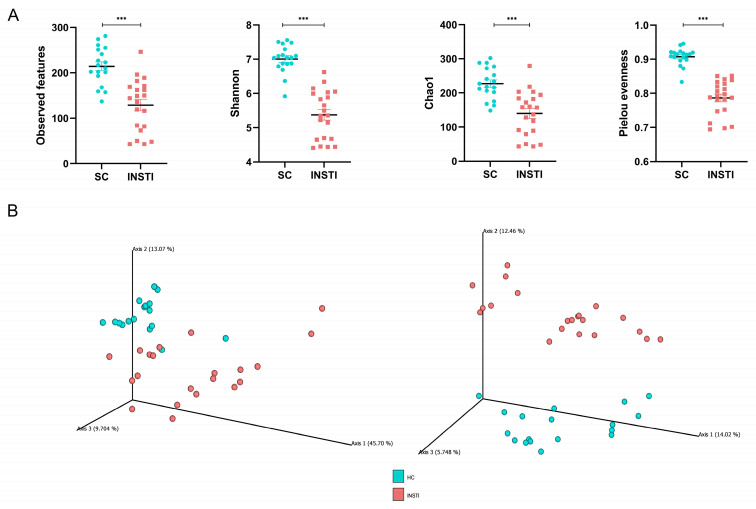
Gut microbiota alpha and beta diversity metrics. (**A**) Observed features, Shannon, Chao1, and Pielou evenness indices of the SC group compared with the INSTI patients. Mann–Whitney U test, *** *p* < 0.001; (**B**) beta diversity plots for weighted (left) and unweighted (right) UniFrac distances in the INSTI group (red) and seronegative control group (turquoise). PERMANOVA-tested, pseudo-F for weighted UniFrac = 9.89, pseudo-F for unweighted UniFrac = 4.89. *p* = 0.001, and *q* = 0.001 in both cases.

Alpha diversity metrics, which account for richness, diversity, and rare species and evenness, were significantly lower in the INSTI group, in comparison with the SC group (Figure 1A). This reflected a clearly dysbiotic gut profile. As sexual behavior is an independent factor that might contribute to the observed GM differences, multiple comparisons among alpha diversity metrics were performed. We did not observe any significant differences between men who had sex with men and heterosexual participants in terms of alpha diversity (Supplementary Table S2).

Principal coordinate analysis for beta diversity metrics also showed significant differences between the two studied groups (Figure 1B, *p* < 0.001), revealing defined clusters in metric distances which considered quantitative (weighted UniFrac) and qualitative (unweighted UniFrac) characteristics. As performed previously, multiple comparisons among beta diversity metrics were performed considering the participants’ sexual behavior. The only significant difference found was between patients under INSTIs (MSM and HTS) through the weighted UniFrac distance. It is notable that this metric weighs quantitative aspects of the observed taxa, rather than qualitative ones. Therefore, it can minimize the weight of less abundant organisms that may be important in the structure of the microbiota (Supplementary Table S3).

### 2.3. Relative Abundances across Different Taxonomic Levels

**Figure 2:**
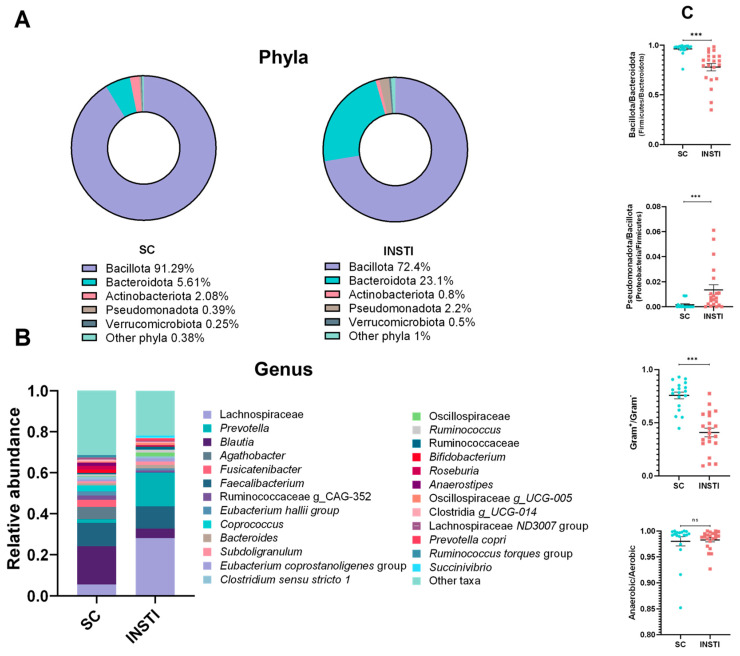
Relative abundances and ratios of different bacteria in the seronegative control group (SC) and in the INSTI group. (**A**) Donut chart representing different phyla in both groups, accompanied by relative abundance percentages. Top 5 phyla are shown, while minor phyla are summarized into the “other phyla” category. (**B**) Stacked barplot of bacterial genera in both groups. Top 25 taxa (ordered by median) are depicted, minor taxa are summarized into the “other taxa” category. (**C**) *Bacillota*/*Bacteroidetes*, *Pseudomonadota*/*Bacillota*, Gram-positive/Gram-negative, and anaerobic/aerobic ratios in the SC and INSTI groups. The ratios were adjusted in such a way that a value close to 1 indicates a greater relative abundance of the bacteria described in the numerator (*Bacillota*, *Pseudomonadota*, Gram-positive, and anaerobic bacteria), while a value close to 0 indicates a greater abundance of the bacteria indicated in the denominator (*Bacteroidota*, *Bacillota*, Gram-negative, and aerobic bacteria). Results are showed as mean ± SEM (standard error of the mean). Comparisons were analyzed using Mann–Whitney test. *** *p* < 0.001, ns = not significant.

We observed a decrease in the relative abundance of the phyla *Bacillota* and *Actinobacteriota* in the INSTI group compared to the SC group (72.4% vs. 91.3 and 0.8% vs. 2.1%, respectively). On the other hand, increased relative abundances were noted in the INSTI group for *Bacteroidota* (23.1% in the INSTI group vs. 5.6% in the SC group), *Pseudomonadota* (formerly *Proteobacteria*, 2.2% in the INSTI group vs. 0.4% in the SC group), and *Verrucomicrobiota* phyla (0.5% in the INSTI group vs. 0.25% in the SC group), as displayed in Figure 2A. For deeper taxonomical levels, increased relative abundances were mainly observed in the *Lachnospiraceae* family (5.5% in the SC group vs. 28% in the INSTI group), and *Prevotella* (1.73% in the SC group vs. 16.4% in the INSTI group), while genera such as *Blautia* and *Fusicatenibacter* (18.6% in the SC group vs. 4.67% in the INSTI group and 3.34% in the SC group vs. 0% in the INSTI group), were less abundant or depleted (Figure 2B).

### 2.4. Gut Microbiome Ratios

In line with the previous results, we showed that the *Bacillota*/*Bacteroidota* (b/B; previously indicated as *Firmicutes*/*Bacteroidota*) ratio was significantly increased in the SC group (*p* < 0.001), reinforcing the abundance of the *Bacillota* phylum with respect to Bacteroidetes species (Figure 2C, top). On the other hand, the *Pseudomonadota*/*Bacillota* (P/b, formerly *Proteobacteria* /*Firmicutes*) ratio was significantly increased in the INSTI group, showing the abundance of *Pseudomonadota* species in this population (Figure 2C). Furthermore, we also calculated the Gram-positive/Gram-negative (G^+^/G^−^) ratio, which exhibited a dramatically increased proportion of Gram-negative species in the GM of the INSTI group in comparison to that of the SC group, which predominantly consisted of Gram-positive bacteria (Figure 2C). No differences in the ratio of anaerobic/aerobic or aerotolerant bacteria were found when comparing the INSTI and SC groups. Furthermore, significant correlations between the hs-CRP levels, a common biomarker of systemic inflammation related to an increase in mortality, and HIV progression [31] and the P/b and G^+^/G^−^ ratios were noted (Supplementary Figure S1).

### 2.5. Differential Abundance Analysis

**Figure 3:**
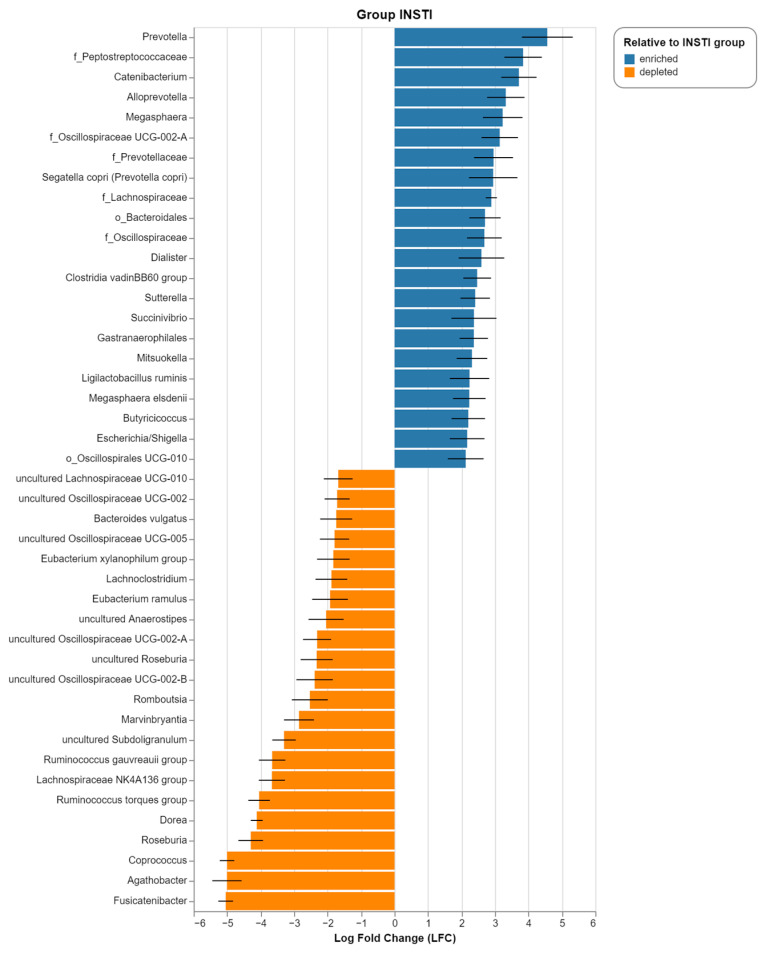
Differentially abundant taxa in the INSTI group. Bars depict ANCOM-BC log fold change (LFC) between the INSTI and SC groups. Top 22 taxa ordered by LFC are shown (LFC ≥ |1.5|). A threshold of *q* < 0.05 was applied. Blue bars represent taxa enriched in the INSTI group, whereas orange bars account for depleted taxa (that is, enriched in the SC group).

To identify differentially abundant taxa in the SC and INSTI groups, we used the ANCOM-BC method. We found that the GM of the INSTI group had significantly greater proportions of *Prevotella*, *Catenibacterium*, *Alloprevotella*, *Segatella copri*, *Dialister*, *Sutterella*, *Ligilactobacillus ruminis*, *Megasphaera elsdenii*, *Butyiricicoccus*, and *Escherichia*/*Shigella*, among others. Conversely, *Fusicatenibacter*, *Agathobacter*, *Coprococcus*, *Roseburia*, *Dorea*, and other taxa were depleted in the INSTI group (and therefore, enriched in the SC group) (Figure 3).

### 2.6. Differential Metabolic Pathways Present in the Gut Microbiota

**Figure 4:**
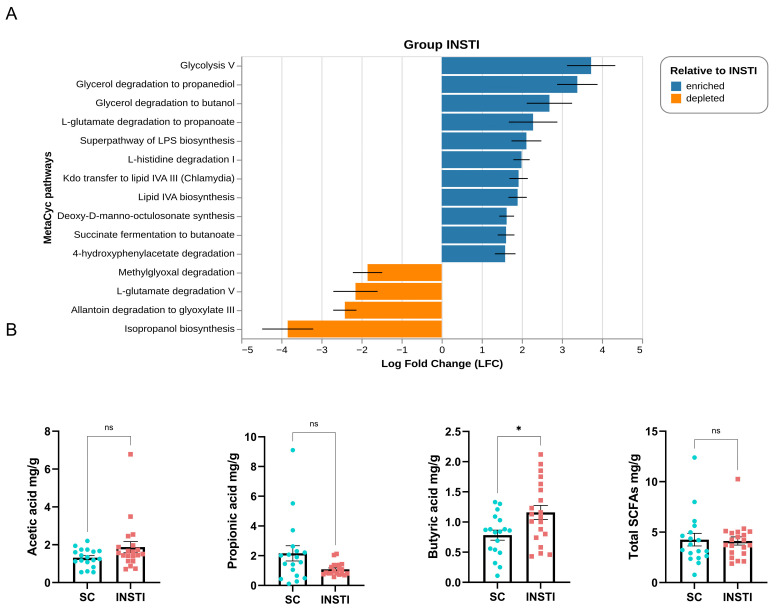
Differential bacterial metabolic pathways obtained by PICRUSt2 and fecal SCFA concentrations in both groups. (**A**) Bars depict ANCOM-BC log fold change between the INSTI and SC groups. Blue bars represent enriched bacterial metabolic pathways (using the MetaCyc database) in the INSTI group, while orange bars show depleted metabolic pathways. Threshold values of *q* < 0.05 and LFC ≥ |1.5| were applied to all PICRUSt2 outputs. (**B**) Fecal SCFA quantification in the seronegative control (SC) and INSTI groups. The figure shows mean concentrations ± SEM of acetic, propionic, butyric acids, and total SCFAs, in terms of mg SCFA/g feces. Comparisons were made by Mann–Whitney U test, * = *p* < 0.05, ns = not significant.

To predict the functional potential of the gut bacterial communities and its differences, we used PICRUSt2 to predict MetaCyc pathways. In the INSTI group, we found that some energy-related pathways such as archaeal glycolysis (P341-PWY), glycerol degradation/utilization and fermentation (GOLPDLCAT-PWY, PWY-7003), as well as histidine degradation (HISDEG-PWY) were characteristic of this group (Figure 4A). Furthermore, we also found that pathways related to the production of different components of bacterial lipopolysaccharides (PWY-1269, PWY-6567, NAGLIPASYN-PWY) as well as to the fermentation of succinate to butyrate (PWY-5677) were also discriminative for the INSTI group. This is very interesting, since the production of butyrate is considered a beneficial bacterial activity. On the other hand, in the SC group, we found pathways related to the degradation of aldehydes and polyamines (METHGLYUT-PWY, PWY-5705). Similarly, we also found pathways related to the production of butyrate; nevertheless, the SC group characteristic pathway was that involving crotonyl-CoA (P162-PWY), which in turn produces acetate via acetyl-CoA, and butyrate via butanoyl-CoA [32].

### 2.7. Fecal SCFA Quantification

Regarding fecal SCFA concentrations, the INSTI group showed a trend of higher acetic and butyric acids fecal concentrations in comparison to the SC group. Notably, butyric acid had a significantly higher concentration (1.08 mg/g feces vs. 0.85 mg/g, *p* < 0.05, Figure 4B). On the other hand, propionic acid had a higher, but not significantly, concentration in the SC group in comparison to the INSTI group (1.73 mg/g vs. 0.86 mg/g, respectively). Total SCFAs did not exhibit notable or significant changes in the two groups. These findings correlate with the PICRUSt2 results regarding differential metabolic pathways related to butyrate in the INSTI group.

## 3. Discussion

### 3.1. ART Is Not Sufficient to Restore the Diversity and Taxonomy of the Gut Microbiota

Different studies indicated that despite its effectivity and the consequent improvement in the quality of life of PLWHIV, ART fails to achieve an adequate restoration of the taxonomic profile of the GM [14,15]. Notwithstanding the effects on the GM, different markers of intestinal damage, bacterial translocation, and the concentration of systemic cytokines have shown an improvement after ART [14,33]. Whether this improvement in the intestinal milieu and are generalizable in different populations remains without a conclusive answer.

Our results showed a clear loss of alpha diversity, both richness and evenness, in PLWHIV compared to seronegative controls. This coincides with previous evidence from the Mexican population [34,35] and different populations [15,36,37], as well as from subjects under different ART regimens or with HIV-associated comorbidities. However, other studies showed contrasting evidence, with non-significant variations in alpha diversity and even increases in some metrics in PLWHIV compared to healthy controls [13,26,38]. Some of these differences may be due to technical factors, such as the metrics used to evaluate alpha diversity, but also to factors inherent to the examined population, since it is known that sociodemographic, cultural, or dietary factors can also exert a strong influence on the intestinal microbiota. Despite this, our beta diversity metrics results indicate that the intestinal microbiota in PLWHIV presents taxonomic profiles that are significantly different from that of the seronegative population, which is in accordance with other studies [27,36,37].

There are different reports that indicate that sexual behavior is a factor that, by itself, influences the composition of the intestinal microbiota [37]. In our study, we observed a distancing in terms of beta diversity between the subgroups of heterosexual (HTS) and MSM (men who have sex with men) PLWHIV, using the weighted UniFrac metric (Supplementary Table S3, pseudo-F = 3.57, *p* = 0.021). However, this was not corroborated by other qualitative metrics [39]; so, we could not decisively conclude that sexual behavior constituted an influential factor in the beta diversity of PLWHIV in our studied population.

### 3.2. An Apparently Mixed Taxonomic Profile Is Shown by PLWHIV under INSTIs

The alterations observed in the relative bacteria abundances in the INSTI group, which mainly comprised decreases in the *Bacillota* phylum, occurred at the expense of increases in *Bacteroidota* and *Pseudomonadota*; this was reflected in the *Bacillota*/*Bacteroidota* (b/B) and *Pseudomonadota*/*Bacillota* (P/b) ratios. A high F/B ratio is considered a potential marker of the predisposition to a more efficient extraction of dietary calories and, consequently, is related to an increase in the catabolic capacity and production of SCFAs and is associated with obesity [40]. Evaluating this potential in PLWHIV through the intestinal microbiota would be interesting in the context of the metabolic alterations related to weight gain, the increase in cardiovascular risk associated with the use of INSTIs, and the switch from TDF (tenofovir disoproxil fumarate) to TAF [30,41]. In the case of our population, this ratio suggested that the INSTI group had a lower caloric extraction capacity through the intestinal microbiota than the seronegative controls. However, this ratio resulted in inconsistent data [40]; moreover, increases in *Bacteroidota* were associated with type 2 diabetes (T2D), cirrhosis, colon cancer, metabolic syndrome (MetS), and non-alcoholic fatty liver disease [42,43]; thus, these findings and their correct interpretation in Mexican PLWHIV require further studies.

On the other hand, the P/b ratio, scarcely reported in the literature, reflects the expansion of bacteria from the *Pseudomonadota* phylum at the expense of *Bacillota*, a trait that agrees with data from Villanueva-Milán et al., who evaluated patients in similar conditions [44]. Interestingly, reanalyzing data previously published by our research group [35,45], it was shown that the P/b ratio increased significantly in PLWHIV with MetS, compared to PLWHIV without MetS (*p* < 0.05, Supplementary Figure S2), demonstrating the strong relationship between this ratio and a pro-inflammatory state of the gut microbiota. Additionally, it is known that a Gram^+^/Gram^−^ ratio < 0.4 could be associated with severe fragility and metabolic endotoxemia; therefore, we evaluated the Gram^+^/Gram^−^ proportion, showing that the INSTI group had a significantly higher proportion of Gram^−^ species than the SC group (mean 0.40 vs. 0.76, *p* < 0.001), indicating an inflammatory condition, probably mediated by the release of LPS or bacterial by-products into the systemic circulation, contributing to establishing or reinforcing an inflammatory state [40].

Examining the taxonomic changes at the family and genus levels, we observed a clear increase in bacteria of the *Lachnospiraceae* family in the INSTI group. This agrees with the differential abundance analysis, which showed a mixed taxonomic profile: on the one hand, SCFA-producing bacteria (*Lachnospiraceae*, *Oscillospiraceae*, *Butyricicoccus*), on the other, those that also have a potentially pathogenic role (*Prevotella* spp., *S. copri*, *Escherichia*/*Shigella*, *Succinivibrio*, *Sutterella*, *Ligilactobacillus ruminis*, *Megasphera elsdenii* [14,46,47,48,49]). Furthermore, there is evidence that *Clostridium immunis* and *Ruminococcus gnavus*, commensal bacteria within the *Lachnospiraceae* family, robustly inhibit HIV infection through tryptophan metabolism by the aromatic amino acid aminotransferase (ArAT) and aryl hydrocarbon receptor (AhR) pathways [50]. Thus, nowadays, we know that HIV infection leads to dysbiosis in the gut, but we are far to know how single species of the gut microbiota could impact HIV infection.

A common taxonomic finding in various studies in PLWHIV is an increase in *Prevotella* species, such as *S*. *copri* [14,34,51,52]. In the Mexican population, this increase was associated with HIV-related comorbidities, such as MetS [35,44]. The comparison with the seronegative population allowed us to observe that the increase in the relative abundance of *Prevotella* (SC group, 1.7% vs. INSTI group, 16.4%) and the significant presence of species such as *S*. *copri* showed the association of these genera with HIV infection, independently of the presence of comorbidities such as MetS and T2D.

Among the other genera that characterized the INSTI group versus the SC group there was, for example, *Succinivibrio*. This is a genus of Gram-negative anaerobic bacteria that was differentially detected in PLWHIV from different populations under virological suppression [35,53]. Interestingly, it was shown that the abundance of this genus decreases significantly in treatment-naïve patients [14]. On the other hand, *Sutterella*, also a Gram-negative bacterium, has received attention due to its role in ulcerative colitis [47]. Interestingly, LPS derived from this species does not induce a potent inflammatory response [54]. However, *Sutterella* can degrade IgA through specific proteases; so, instead of directly inducing inflammation, this bacterium could be involved in altering the functionality of the antibacterial response at the intestinal level [47].

Likewise, we found that *L. ruminis* also significantly characterized the intestinal microbiota of the INSTI group. This species is one of the few within the *Lactobacillus* genus that possess flagellins, an immunogenic factor that confers motility and promotes monocytic hyperactivation, favoring a constant production of proinflammatory cytokines in PLWHIV through its main ligand, TLR5 [55].

Though it was demonstrated that *L*. *ruminis* flagellins do not seem to elicit an inflammatory response as high as that induced by flagellins derived from other species, such as *Salmonella typhimurium* or *Listeria monocytogenes* [56], other findings are intriguing. For example, Yamashiro et al. found significantly higher abundances of *L. ruminis* in the intestinal microbiota of patients with ischemic stroke [57]. In line with this, in vitro experiments determined that flagellins derived from *L*. *ruminis* induced a greater expression of thromboplastin in monocytes from PLWHIV [58]. These findings are important, given that PLWHIV, even under virological suppression, have a significantly higher risk of ischemic stroke [59].

In correlation with the significant elevations in fecal butyrate, we found differentially abundant butyrogenic species in the INSTI population. Among them, *Megasphaera elsdenii* stood out, belonging to the class *Negativicutes* and the phylum *Bacillota* [60]. The role of *M*. *elsdenii* in the intestinal microbiota of PLWHIV is poorly known; however, it was shown that this bacterium is able to activate dendritic cells and induce cytokine production and is associated with a higher risk of contracting HIV [24,49]. Interestingly, in vitro studies showed that the co-culture of *Lactobacillus acidophilus* with *M*. *elsdenii* induced an overproduction of butyrate [24].

Considering this, we performed co-occurrence network analyses (Supplementary Figure S3), which, in PLWHIV, showed a positive correlation of *Megasphaera* with *Escherichia*/*Shigella* (R = 0.72, *p* < 0.01), as well as with *Faecalibacterium* and *Phascolarctobacterium*, these last described as SCFA-producing bacteria [61]. These results agree with a study in the Chinese population, which described a network of *Faecalibacterium*, *Phascolarctobacterium*, *Butyricicoccus*, and *Prevotella* that correlated with increases in serum TNF-α and IL-6 in PLWHIV [26]. Finally, *M*. *elsdenii* also seems to have implications in the vaginal microbiota, constituting a marker associated with the risk of acquiring HIV in sex workers in the African population [62].

Another significant butyrogenic genus in our studied population is *Butyricicoccus*. In addition to constituting a characteristic genus in PLWHIV in the Chinese population [26], it was reported in the Spanish population under virological suppression with >250 CD4^+^ T cells/μL [52], as well as in PLWHIV with obesity [63]. Interestingly, this last study reported a depletion of *Butyricicoccus* in healthy-weight patients and seronegative controls. Complementary to the above, studies in Rhesus macaques infected with SIV that started ART (tenofovir, emtricitabine, and raltegravir), showed a significant increase in the abundance of *Butyricicoccus*, a trait absent in untreated macaques [64]. Our results indicate that *Butyricicoccus* constitutes a statistically significant genus in PLWHIV with INSTI-based treatment under effective virological suppression. Notably, we observed that, in our cohort, the abundance of *Butyricicoccus* was positively correlated with the number of years on ART, once controlling for chronological age (Spearman partial correlation ρ = 0.451, *p* = 0.046).

These findings clearly show that the gut microbiota of PLWHIV in western Mexico is characterized by an environment enriched in butyrogenic and other potentially pathogenic bacteria, a trait that was observed in other patient populations in Latin America [27]. The role of apparently butyrogenic species, especially those catalogued within the family *Lachnospiraceae*, in PLWHIV should be investigated in view of the apparent increase in their relative abundances compared to the seronegative population, even under virological suppression in the absence of other significant comorbidities.

### 3.3. Alterations in the SCFA Profile Correlate with a Distinct Butyrogenic Gut Microbiota in PLWHIV

Consistent with the described taxonomic profile, we observed that the profile of fecal SCFAs reflected a microbiota that conserved certain functional aspects, such as butyrate production. It is known that the main anti-inflammatory effect of SCFAs, primarily butyrate, is due to their activity as pan-inhibitors of HDACs, inducing structural changes in chromatin [21]. This mechanism has served as the basis of novel strategies for eliminating the viral reservoir [65]. In this sense, it is possible that proviral DNA that persists in infected cells despite ART, which may also reside episomally, is reintegrated into nucleosomes and favors a successful viral replication through exposure to butyrate [65,66,67], as well as increasing the infection and replicative capacity of the virus in THP1 monocytes [68]. This epigenetic stimulus is also capable of reducing the expression of genes in response to type 1 interferon (antiviral IFN-stimulated genes, ISGs) in HT-29 colon cells [68]. It is interesting to note that during influenza infection, exposure to butyrate increases the number of viral particles, though significantly alleviating the tissue damage mediated by the IFN-1 response [69]. In view of these findings, some authors suggested that a butyrogenic environment could play a role in the possible reactivation of latent HIV-1 in infected cells [70]. It was widely demonstrated that SCFAs have a generally positive effect on the intestinal milieu due to their anti-inflammatory effects; however, this evidence suggests that these effects may be different in PLWHIV.

Our results indicate that the production of butyrate in the GM in the INSTI group occurs through the fermentation of succinate towards butyrate, a poorly described metabolic pathway, since the “classical” metabolic pathway of butyrate production is through the fermentation of acetyl-CoA [17]. Other commensal genera of the intestinal microbiota, such as *Prevotella*, *Bacteroides*, or *Veillonella*, metabolize non-digestible carbohydrates to propionate via the succinate pathway [71]. However, there is no evidence that *Prevotella* spp. or *S*. *copri*, significantly identified in the INSTI group, can produce butyrate [72].

With the purpose of inferring the taxa that could be responsible for butyrate production in the INSTI group, a BLASTn search was performed considering the differentially abundant taxa containing homologs of butyryl-CoA:acetate CoA-transferase, 4-hydroxybutyrate CoA-transferase, and butyrate kinase, key enzymes in the biosynthesis of butyrate from acetyl-CoA, succinate, and butyryl-CoA, respectively. We found that both *Megasphaera* and *Butyricicoccus* were the only taxa that possess the enzymatic machinery to produce butyrate through the succinate (PWY-5677) and acetyl-CoA condensation pathways (Supplementary Table S4). Notably, *Prevotella* does not have the intracellular machinery for butyrate production, although it has the capacity to produce propionate and succinate [73,74]. These findings deepened the description of the observed bacterial metabolism, indicating that in addition to specific metabolic pathways in the butyrogenic microbiota of PLWHIV (such as the succinate pathway), genera such as *Megasphaera* and *Butyricicoccus* are presumably responsible for butyrate production, at least partially. This butyrogenic profile, enriched in *Lachnospiracaeae* and in *Pseudomonadota*, is interesting, given that it was observed that in patients with HIV, the ability to produce IgA/IgG against neo-antigens derived from *Bacillota* and *Pseudomonadota* is significantly decreased [75,76].

In addition to the different taxonomic profile observed, other mechanisms could explain the observed increase in butyrate, such as possible alterations in the intestinal epithelium. Data from the transcriptome of T CD4^+^ intestinal cells exposed to HIV-1 and *Prevotella* infection [77] showed significant alterations in the expression of the transporter SLC16A1, a key protein in butyrate transport [78]. Decreases in the expression of this protein showed that it impaired the oxidation of butyrate by enterocytes [79], which in turn was related to the loss of physiological levels of hypoxia and favored an increase in aerotolerant and pro-inflammatory species [25]. Evidence of the importance of physiological levels of hypoxia in HIV infection comes from a study by Buccigrossi et al. [80], who demonstrated that the enterotoxic effect of the non-structural HIV protein Tat is abolished through the administration of N-acetylcysteine. This is an important finding, because non-structural proteins maintain their synthesis despite ART [81,82]. The precise relationships between the increase in aerotolerant species, such as those belonging to the phylum *Pseudomonadota*, probable alterations in the absorption of butyrate, and oxidative stress, as well as therapeutic alternatives based on the redox balance at the intestinal level, require to be studied thoroughly, as bases of therapies aimed at reducing dysbiosis.

As previously described, the role of butyrate is still hard to comprehend. In vitro evidence from Caco-2 cells demonstrated that butyrate at concentrations higher than 5 mM can induce intrinsic apoptosis via the upregulation of proapoptotic proteins such as Bak, accompanied by reduced levels of the antiapoptotic protein Bcl-xL [83]. Whether this effect might influence gut permeability and induce low-grade endotoxemia in PLWHIV on an ART regimen remains to be investigated. Additionally, recent in vitro evidence showed that butyrate and a butyrogenic GM can worse the inflammatory process in colitis through an NLRP3 inflammasome-dependent process [84]. This is interesting, since such changes are accompanied by elevated levels of *Pseudomonadota*, a finding in line with those we presented in this work.

This study has some limitations. Due to its cross-sectional design, it was impossible to infer causality from the events described. Additionally, this was a single-center study in the western Mexican population. Due to the multifactorial nature of the changes in the gut microbiota, the results cannot be fully generalized to patients from other geographic areas or other ethnicities. Another important factor is that our seronegative group consisted of self-declared heterosexual participants. In addition, there were differences in the use of tobacco, alcohol, and comorbidities, which may have influenced the composition of the GM. Nevertheless, one of the main strengths of this work is the adequate selection of PLWHIV, who received the current first-line ART recommended regimen, evaluated with robust bioinformatic analysis, including methods based on compositional mathematics, which reduce the risk of bias and false positives. Also, we corroborated our functional predictions using standard methodologies, such as gas chromatography.

## 4. Materials and Methods

### 4.1. Design of the Study and Recruited Participants

Cross-sectional observational study including 21 PLWHIV from the HIV Unit of the Hospital Civil, Guadalajara, Jalisco, Mexico (western Mexico), and 18 seronegative controls (SCs), recruited from January 2019 to May 2021. PLWHIV were receiving an INSTI-containing regimen (INSTI group). The Hospital Ethics Committee approved the study (n.061/19 and n.010/20). The SC group was recruited from the same community. Written informed consent was obtained from each participant prior recruiting. All the guidelines from the Helsinki Declaration (1975, as revised in Brazil 2013) were followed. For the recruiting of the INSTI group, the criteria were as follows: adults (18–60 years) with HIV infection for at least one year with an undetectable HIV viral load for at least six months. The exclusion criteria for enrollment were as follows: pregnant or lactating women, active AIDS-defining infection, hepatitis B or C virus infection, glomerular filtration rate < 60 mL/min/1.73 m^2^, chronic pancreatitis, celiac disease, malabsorption syndrome, or metabolic comorbidities. The exclusion criteria included the use of antibiotics, prebiotics, probiotics, immunosuppressants, corticosteroids, non-steroidal anti-inflammatory drugs, vitamins, minerals, or antioxidants within 30 days prior to fecal sample acquisition. The SC group inclusion criteria are detailed in Supplementary Materials.

### 4.2. DNA Extraction from Fecal Samples and 16S rRNA Amplicon Sequencing

Fecal samples were collected and immediately stored at −80 °C. DNA was extracted from the samples using commercial kits: the QIAamp PowerFecal DNA Kit (QIAGEN, Hilden, Germany) and the Quick-DNA Fecal/Soil Microbe Miniprep Kit (Zymo Research, Irvine, CA, USA). The 16S rRNA amplicon sequencing library preparation was performed as described previously [31]. The V3 and V4 regions of the 16S rRNA gene were amplified with Platinum Taq DNA Polymerase High Fidelity (Invitrogen, Waltham, MA, USA). PCR was performed following the manufacturer’s protocol, and the amplicons were further purified with AMPure XP^®^ (Beckman Coulter, Indianapolis, IN, USA) magnetic beads and quantified with the Qubit^®^ 3 dsDNA HS kit (Invitrogen, Waltham, MA, USA) according to manufacturer’s indications. Index incorporation was achieved with the Nextera XT Index Kit v2 Set A (No. Cat. FC-131-2001, Illumina, San Diego, CA, USA). Finally, indexed amplicons were pooled to equimolar concentrations into a 4 nmol/L solution tube. The resulted library was denatured using the MiSeq Reagent V3 600-cycle, Illumina, San Diego, CA, USA).

### 4.3. Bioinformatic Analyses

The main analyses were performed with QIIME2 version 2023.2 [85]. The raw sequence data were filtered by denoising with DADA2 [86]. The resulting amplicon sequence variants (ASVs or features) were assigned to their corresponding taxonomy using a Bayes classifier [87] that was trained on the Silva 138 database [88,89]. Then, ASVs identified as mitochondria and chloroplasts were removed, and the filtered ASVs were aligned with MAFFT [90] and used to construct a phylogeny with FastTree2 [91]. Alpha diversity metrics (Observed features, Chao1, Shannon, and Pielou) were computed. Beta diversity metrics (weighted and unweighted UniFrac distances) and PCoA were generated with the QIIME2 Emperor tool version 2021.8 [92].

Differential abundance analysis of the taxa was performed with ANCOM-BC [93] using the *ancombc* plugin in QIIME2. ANCOM-BC is a compositional statistical method that accounts for sampling fraction and normalize read counts, while controlling for false discovery rates. The false discovery rate (FDR) method incorporated in the ANCOM-BC plugin was used to correct the *p*-values for multiple testing (*q*-value). A *q* < 0.05 cut-off was used to assess significance, and a log fold change (LFC) ≥ |1.5| to assess the effect size.

The Phylogenetic Investigation of Communities by Reconstruction of Unobserved States (PICRUSt2) pipeline [94,95,96,97,98] was used to predict the functional pathways of each group using the MetaCyc Database [99]. The ANCOM-BC method was also employed to analyze PICRUSt2 outputs to determine differentially abundant pathways in the studied groups. A *q* < 0.05 cut-off was used to assess significance, and an LFC ≥ |1.5| to assess the effect size. The *Bacillota*/*Bacteroidetes*, *Pseudomonadota*/*Bacillota*, Gram-positive/Gram-negative, and anaerobic/aerobic bacteria ratio calculations are described in Supplementary Materials (Supplementary Equations). Co-occurrence networks were constructed using SECOM (Pearson2) through MicrobiomeAnalyst 2.0 server [100]. SECOM is a compositional statistical method as well [101], accounting for both sample- and taxon-specific biases.

### 4.4. Fecal Short-Chain Fatty Acid Quantification

The quantification of SCFAs was performed as previously described [101]. Briefly, the fecal samples were immediately frozen after collection and kept at −80 °C until the analysis. Then, 20 mg of feces was transferred to 200 µL of acidified N-butanol, tetrahydrofuran, and acetonitrile (50:30:20), centrifuged, and filtered through a 0.22 µm filter (Whatman GD/X, Merck, Darmstadt, Germany). Next, 3 µL of the filtrate was injected into a Shimadzu gas chromatograph (Shimadzu Scientific Instruments, Kyoto, Japan). The concentrations of SCFAs in the fecal samples were normalized to the wet weight of the feces.

### 4.5. Statistical Analyses

Data normality was examined using the Shapiro–Wilk test. For parametric data, unpaired Student’s *t*-test or one-way ANOVA was performed to assess the differences between the groups; if a non-parametric behavior was observed, the Mann–Whitney U or Kruskal–Wallis test was performed. Fisher’s exact test was applied to evaluate categorical variables. Beta diversity metrics among the groups were compared by performing PERMANOVA tests. Both alpha and beta diversity statistical analyses were corrected with Benjamini–Hochberg (BH) multiple testing. All statistical tests were two-tailed unless stated otherwise, and a *p* or *q*-value ≤ 0.05 was considered statistically significant. The data were analyzed using SPSS 25.0, unless otherwise specified. Plots were generated utilizing GraphPad Prism version 8.0.2.

## 5. Conclusions

We found that the gut microbiota of Mexican PLWHIV under INSTI-based ART, with virological suppression and immunocompetence, was characterized by a significant dysbiosis in terms of structure (alpha and beta diversities) and taxonomic composition, in comparison to that of seronegative healthy controls from the same community. More importantly, a conservation of butyrogenic functions was observed, although with significantly different structure and taxonomies, including the families *Lachnospiraceae* and *Oscillospiraceae*, which encompass butyrogenic bacteria depleted in the seronegative population, such as *Megasphaera elsdenii* and *Megasphaera* spp. and *Butyricicoccus*. This observation is probably related to the significant increase in fecal butyrate concentrations. These changes occurred alongside higher proportions of *Pseudomonadota* and Gram-negative species, such as *Prevotella*, *Escherichia*/*Shigella*, and *L. ruminis*, and the significant enrichment of LPS-producing metabolic pathways. The role of distinct butyrogenic bacteria in the gut microbiota in PLWHIV with chronic infection should be investigated in prospective studies. The use of butyrate-based supplements in PLWHIV should be carefully evaluated. Likewise, therapies based on the modulation of the intestinal milieu conditions, such as antioxidants, could be considered to positively modify the gut microbiota.

## Figures and Tables

**Table 1:** Clinical characteristics of the recruited participants.

Characteristic	SC (*n* = 18)	INSTI (*n* = 21)	*p*-Value
Age (years)	48.72 ± 8.63	44.64 ± 8.33	0.179 ^a^
Sex *n* (%)			
Male	11 (61.1%)	14 (66.7%)	0.750 ^b^
Female	7 (38.89%)	7 (33.3%)	
BMI (kg/m^2^)	25.85 ± 2.85	24.85 ± 3.35	0.310 ^a^
Comorbidities *n* (%)	0 (0%)	7 (33.3%)	0.010 ^b^
Time since HIV diagnosis (years)	NA	7.76 ± 5.26	NA
Duration of ART (years)	NA	7.19 ± 4.83	NA
Time of INSTI use (years)	NA	1.00 (1–2)	NA
INSTI *n* (%)			
Bictegravir (BIC)	NA	18 (85.7%)	NA
Dolutegravir (DTG)		3 (14.3%)	
INSTI-based ART including TAF *n* (%)	NA	18 (85.7%)	NA
ART prior to INSTIs *n* (%)	NA	15 (71.4%)	NA
HIV-1 RNA viral load (copies/mL)	NA	40 (20–40)	NA
Undetectable HIV-1 RNA (≤40 copies/mL)	NA	20 (95.2%)	NA
Low-level viremia (41–199 copies/mL)		1 (4.8%)	
Absolute T CD4^+^ count (cells/μL)	ND	552 (397–718)	NA
Complete immune response (≥500 T CD4^+^ cells/µL) *n* (%)			
Complete	NA	11 (52.4%)	NA
Incomplete		10 (47.6%)	
Nadir T CD4^+^ count (cells/μL)	NA	243 (109–401)	NA

Abbreviations: SC: seronegative controls, INSTI: integrase strand-transfer inhibitor group, BMI: body mass index, ND: not determined, NA: not applicable, TAF: tenofovir alafenamide. Data are showed as mean ± standard deviation or median with interquartile range in parentheses. *p* values were calculated by (^a^) Student’s *t*-test or (^b^) Fisher’s exact test.

## Data Availability

The data that support the findings of this study are available from the corresponding authors upon reasonable request.

## Supplementary Materials

The following supporting information can be downloaded at: https://www.mdpi.com/article/10.3390/ijms25094830/s1. References [1,15,25,93,102,103,104] are cited in the Supplementary Materials.
