# Supplementary material for: A Two-Faced Gut Microbiome: Butyrogenic and Proinflammatory Bacteria Predominate in the Intestinal Milieu of People Living with HIV from Western Mexico
Source: Int J Mol Sci. 2024 Apr 29;25(9):4830. doi: 10.3390/ijms25094830 (PMC11084381; doi:10.3390/ijms25094830)
Supplement: Supplementary file 1 [file ijms-25-04830-s001.zip › ijms-2917322-supplementary.pdf]

Supplementary material: A two-faced gut microbiome: butyrogenic and proinflammatory bacteria predominate in the intestinal milieu of people living with HIV from western Mexico

Tonatiuh Abimael Baltazar-Díaz[1,2], Jaime F. Andrade-Villanueva[2,3], Paulina Sánchez-Álvarez[3], Fernando Amador-Lara[3], Tania Holguín-Aguirre[3], Karina Sánchez-Reyes[2], Monserrat Álvarez-Zavala[2], Rocío Ivette López-Roa[4], Miriam Ruth Bueno-Topete*[1], and Luz Alicia González-Hernández*[2,3]

[1]Instituto de Investigación en Enfermedades Crónico Degenerativas, Universidad de Guadalajara
[2]Instituto de Investigación en Inmunodeficiencias y VIH, Universidad de Guadalajara
[3]Unidad de VIH, Hospital Civil de Guadalajara, Unidad Hospitalaria Fray Antonio Alcalde
[4]Laboratorio de Investigación y Desarrollo Farmacéutico, Universidad de Guadalajara

1 Supplementary information S1: Criteria for seronegative healthy controls (SC group)

Inclusion criteria:

- Adults (18-60 years)
- BMI between 18.5 and 29.9 kg/m^2
- No known allergies or intolerance to fibre sources
- No gastrointestinal surgery
- No history of chronic gastrointestinal disorders (Crohn's, ulcerative colitis, IBD), type I or type II diabetes, autoimmune diseases, or cancer
- Non-vegetarian or smokers
- Alcohol intake $\leq$ 28 g/week

Exclusion criteria:

- Positive serologies for HIV-1/HIV-2, hepatitis B, hepatitis C or syphilis.
- Use of prebiotics or probiotics 30 days before recruiting
- Use of antibiotics 90 days before recruiting
- Use of statins, statins, immunosuppressants, corticosteroids, non-steroidal anti-inflammatory drugs, vitamins, minerals or antioxidants within 30 days prior to recruiting.

2 Supplementary Table S1

Clinical and biochemical parameters of participants

	SC n=18	INSTIs n=21	*p*-value
Current smoking n (%)	0 (0%)	7 (33.3%)	0.010 [c]
Current alcohol n (%)	0 (0%)	13 (61.9%)	0.000 [c]
Illicit drug use n (%)	0 (0%)	0 (0%)	NA
Comorbidities			
	18 (100%)	14 (66.7%)	
None n (%)			
	0 (0%)	6 (28.6%)	0.026 [c]
Osteoporosis n (%)			
	0 (0%)	1 (4.8%)	
Borderline personality disorder n (%)			
Sexual behaviour			
	18 (100%)	10 (47.6%)	
HTS n (%)			0.000 [c]
	0 (0%)	11 (52.4%)	
MSM n (%)			
Haemoglobin g/dL	15.1 ± 0.9	15.22 ± 1.60	0.778 [a]
Platelets 10^3/µL	229.59 ± 63.50	235.41 ± 81.64	0.807 [a]
Leukocytes 10^3/µL	5.68 ± 1.41	4.89 ± 1.66	0.127 [a]
Urea mg/dL	33.36 ± 7.60	32.0 ± 7.94	0.604 [a]
Creatinine mg/dL	0.82 ± 0.122	0.89 ± 0.18	0.160 [a]
GFR (mL/min/1.73 m^2)	102.6 (94.95-109.35)	101.40 (75.90-112.60)	0.542 [b]
GGT UI/L	23.50 (15.25-36.25)	26.0 (20.0-36.50)	0.426 [b]
ALP UI/L	69.67 ± 22.21	79.61 ± 20.88	0.158 [a]
AST UI/L	20.00 (15.0-30.0)	28.00 (20.0-37.50)	0.049 [b]
ALT UI/L	26.00 (14.25- 38.75)	23.0 (19.0-37.0)	0.770 [b]
TB mg/dL	0.65 (0.58- 0.8)	0.62 (0.48-0.87)	0.666 [b]
DB mg/dL	0.10 (0.1-0-2)	0.11 (0.09-0.19)	0.917 [b]
Total protein g/dL	7.16 ± 0.47	7.25 ± 0.38	0.480 [a]
Albumin g/dL	4.47 ± 0.32	4.44 ± 0.288	0.860 [a]
Glucose mg/dL	88.5 ± 9.49	89.23 ± 7.53	0.788 [a]
Insulin µU/mL	ND	7.66 ± 3.57	NA
HOMA-IR	ND	1.56 (1.13-2.08)	NA
Triglycerides mg/dL	152.5 ± 100.14	125.10 ± 49.58	0.312 [a]
Total cholesterol mg/dL	217.5 ± 51.02	187.90 ± 38.38	0.082 [a]
HDL-C mg/dL	48.1 ± 13.31	39.19 ± 7.08	0.025 [a]
LDL-C mg/dL	130.6 ± 43.97	122.61 ± 35.15	0.590 [a]
VLDL mg/dL	30.6 ± 20.07	24.80 ± 10.03	0.289 [a]
Triglycerides/HDL ratio	3.43 ± 2.35	3.41 ± 1.74	0.979 [a]
hs-CRP mg/L	2.05 (1.04-3.41)	1.60 (1.05-3.15)	0.512 [b]

Abbreviations: HTS: Heterosexual, MSM: Men who have Sex with Men, GFR: Glomeru-lar filtration rate, GGT: Gamma-glutamyl transferase, ALP: Alkaline phosphatase, AST: Aspartate transaminase, ALT: Alanine transaminase, TB: Total bilirubin, DB: Direct bilirubin, HOMA-IR, HDL-C: High-density lipoprotein cholesterol, LDL-C: Low-density lipoprotein cholesterol, VLDL: Very low-density lipoprotein, hs-CRP: High-sensitivity C-reactive protein, ND: Not determined, NA: Not applicable. Data are shown number of cases and percentage (n %), median and interquartile range (if non-parametric) or mean ± standard deviation (if parametric). *p* values were calculated by ([a]) Student's T test, ([b]) Mann-Whitney or ([c]) Fisher's exact test.

3 Supplementary Table S2

Multiple comparisons in evaluated alpha diversity metrics for sexual behaviour

Group 1	Group 2	H	p-value	q-value
ACE				
MSM_INSTI	HTS	12.61	0.00	0.00
MSM_INSTI	HTS_INSTI	0.01	0.92	0.92
HTS	HTS_INSTI	8.00	0.00	0.01
Pielou				
MSM_INSTI	HTS	18.23	0.00	0.00
MSM_INSTI	HTS_INSTI	0.50	0.48	0.48
HTS	HTS_INSTI	18.62	0.00	0.00
Observed features				
MSM_INSTI	HTS	13.59	0.00	0.00
MSM_INSTI	HTS_INSTI	0.00	0.94	0.94
HTS	HTS_INSTI	9.42	0.00	0.00
Chao1				
MSM_INSTI	HTS	0.00	12.61	0.00
MSM_INSTI	HTS_INSTI	0.92	0.01	0.92
HTS	HTS_INSTI	0.01	7.87	0.01
Faith				
MSM_INSTI	HTS	0.05	0.82	0.92
MSM_INSTI	HTS_INSTI	0.02	0.89	0.92
HTS	HTS_INSTI	0.01	0.92	0.92
Shannon				
MSM_INSTI	HTS	18.23	0.00	0.00
MSM_INSTI	HTS_INSTI	0.12	0.72	0.72
HTS	HTS_INSTI	17.40	0.00	0.00

Abbreviations: MSM INSTI: Men who have sex living with HIV (n=11), HTS INSTI: Het-erosexual living with HIV (n=10), HTS: Seronegative heterosexual (n=18). p and q values were calculated by Kruskal-Wallis test with Benjamini-Krieger-Yekutieli multiple testing.

4 Supplementary Table S3

Multiple comparisons in beta diversity metrics for sexual behaviour.

Group 1	Group 2	Sample size	Pseudo-F	p-value	q-value
Weighted Unifrac distance					
MSM_INSTI	HTS	29	13.85	0.001	0.0015
MSM_INSTI	HTS_INSTI	21	3.57	0.021	0.021
HTS	HTS_INSTI	28	6.07	0.001	0.0015
Unweighted Unifrac distance					
MSM_INSTI	HTS	29	4.25	0.001	0.0015
MSM_INSTI	HTS_INSTI	21	1.36	0.14	0.14
HTS	HTS_INSTI	28	3.10	0.001	0.0015

Abbreviations: MSM INSTI: Men who have sex living with HIV, HTS INSTI: Heterosexual living with HIV, HTS: Seronegative heterosexual. p and q values were calculated by PERMANOVA test with Benjamini–Hochberg multiple testing. Permutations in all cases: 999.

5 Supplementary Equations

Supplementary equations 1 and 2. *Bacillota/Bacteroidetes* (formerly *Firmicutes/Bacteroidetes*), *Pseudomonadota/Bacillota* (previously known as *Proteobacteria/Firmicutes*), $\text{Gram}^{+}/\text{Gram}^{-}$ and anaerobic/aerobic ratios: After centred log-ratio (clr) transformation, as described in bioinformatic methods section, the following equations were applied to relative abundance transformed data at phylum (equations 1 and 2) as previously published [15] and genus or species level (equation 3).

For Gram-Positive/Gram-Negative ratio, taxa were filtered from raw ASV table collapsed at the species level, using the following criteria: All those not identified at the genus or species level were removed, as well as those with putative names (that is, an invalid Full Scientific Name PNU, numbers in the name, etc.), uncultured species, or without clear taxonomic assignment. Next, with the filtered taxa (n=282), we examined their Gram staining according to the BacDive database [102]. Undetermined or variable Gram staining taxa were removed. Given the case that the genus or specie were not in the BacDive database, the primary publication of each genus or specie was examined. With this, a Gram-categorized taxa table was constructed per subject, to later use equation 3.

To evaluate the oxygen tolerance of the taxa found, we used the BacDive database as well; in order to expand our search, we refer to the methodology described by Dubourg et al. [25] using public data available (https://www.mediterranee-infection.com/wp-content/uploads/2020/05/OXYTOL-1.3.xlsx). Given the case that the genus or specie were not in the aforementioned databases, the primary publication of each genus or specie was examined. Using an ASV table collapsed at the species level, putative or invalid taxa, without clear taxonomy, as well as undetermined oxygen tolerance parameters were removed (n=285 after filtering). Obligate aerobes, microaerophiles, and facultative anaerobes were considered aerobes, whereas only obligate anaerobes were considered as true anaerobes, according to Panigrahi et al [103]. With this, an oxygen tolerance table was constructed per subject, to later use equation 4.

$$Bacillota/Bacteroidetes = Bacillota/\left(Bacillota + Bacteroidetes\right)$$

$$Pseudomonadota/Bacillota = Pseudomonadota/\left(Pseudomonadota + Bacillota\right)$$

$$Gram^{+}/Gram^{-} = Gram^{+}/\left(Gram^{+} + Gram^{-}\right)$$

$$Anaerobic/Aerobic = Anaerobic\ taxa/\left(Anaerobic\ taxa + Aerobic\ taxa\right)$$

6 Supplementary Figures

6.1 Supplementary Figure S1

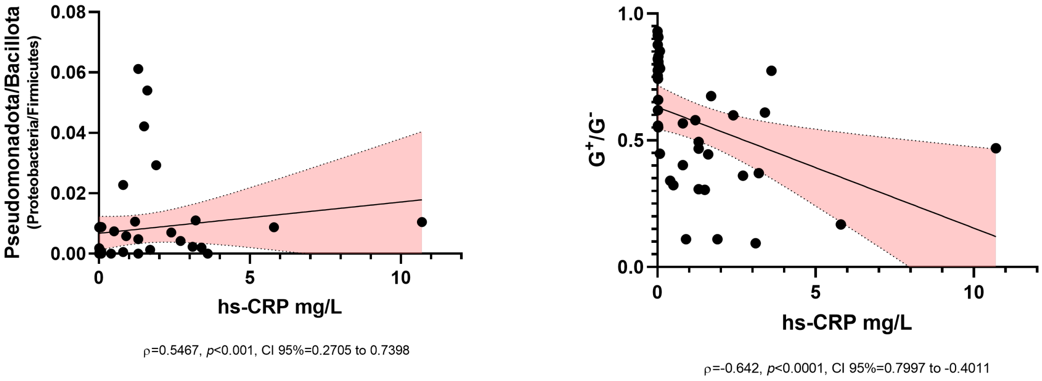

Figure S1. Correlations between hs-CRP and *Pseudomonadota/Bacillota* and Gram-positive/ Gram-negative ratios. Spearman's rho, *p*-values (two-tailed) and confidence intervals (CI) are showed below each diagram.

6.2 Supplementary Figure S2

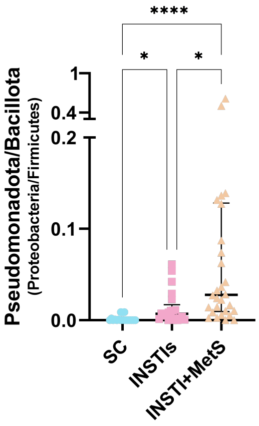

Figure S2. Scatter plot of *Pseudomonadota/Bacillota* (P/b) ratio in seronegative controls (SC), INSTIs (PLWHIV with INSTI-based ART) and INSTI+MetS (PLWHIV-INSTI plus metabolic syndrome). Data from INSTI+MetS are from a previously published study [1]. A significant increase in P/F ratio in INSTI+MetS group is appreciated. Results are showed as mean ± SEM. Analysed by Kruskal-Wallis test with BH multiple testing correction. * $p < 0.05$, **** $p < 0.0001$

6.3 Supplementary Figure S3

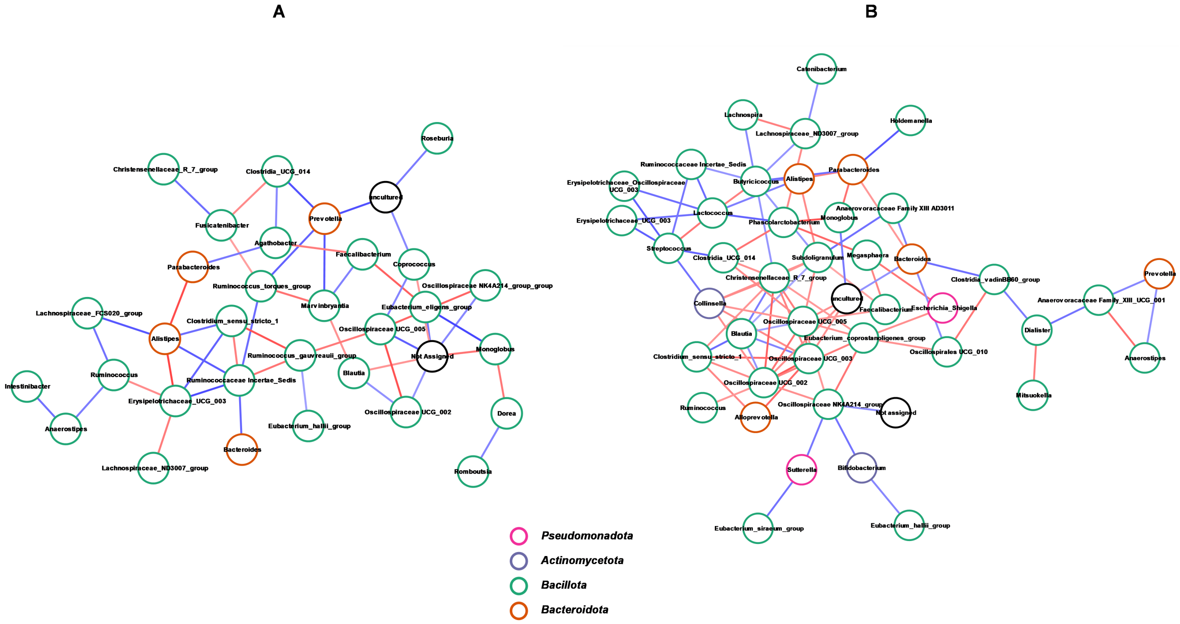

Figure S3. Correlation networks analysis of SC (**A**) and PLWHIV (**B**) group at genus and family level. Correlations were calculated using SECOM [93] with a coefficient threshold $\rho > 0.6$ and a p-value < 0.05. Graph were generated through Cytoscape. Red lines represent positive correlations, whereas blue lines represent negative correlations. Colour circles indicate the corresponding phyla.

7 Supplementary Table S4

BLASTn screening We performed a screening for the presence or absence of butyrate-producers critical enzymes focused on the differentially abundant taxa identified in the INSTI group, excluding taxa not identified at the genus or species level, except members of the *Lachnospiraceae* family. (enlisted below). The search for homologs was based on the dicontiguous megablast and blastn strategies, using the listed query sequences and the NCBI taxonomic annotation, against standard databases. All BLAST searches were performed using an E-Value cut-off of 10^{-10}.

Differentially abundant taxa:

Prevotella	*Lachnospiraceae*	*Mitsuokella*
Catenibacterium	*Segatella copri*	*Ligilactobacillus ruminis*
Alloprevotella	*Dialister*	*Butyricicoccus*
Megasphaera sp.	*Sutterella*	*Escherichia*
M. elsdenii	*Succinivibrio*	*Shigella*

Acetyl-CoA fermentation to butanoate (PWY-5676) homologs

Species	NCBI Reference Sequence	Genome Position
Butyricicoccus faecihominis	NZ_BLYJ01000023.1	29940-31280
Clostridium kluyveri	NC_009706.1	3646071-3647384
Faecalibacterium prausnitzii	NZ_DS483503.1	313586-314932
Roseburia intestinalis	NZ_LR027880.1	431769-430426
Succinate fermentation to butanoate (PWY-5677) homologs		
Clostridium aminobutyricum	AJ250267.3	1093-2574
Clostridioides difficile	GENBANK: NZ_BIWN01000063.1	16750-18246
Butyrate kinase pathway [104] homologs		
Coprococcus eutactus	NZ_CP085930.1	310121-309054
